# How I optimize power to avoid VILI

**DOI:** 10.1186/s13054-019-2638-8

**Published:** 2019-10-21

**Authors:** John J. Marini

**Affiliations:** Division of Pulmonary and Critical Care Medicine, Regions Hospital, University of Minnesota, MS11203B, 640 Jackson St., Minneapolis/ St. Paul, MN USA

**Keywords:** Ventilator-induced lung injury, VILI, ARDS, Energy, Power, Mechanical ventilation

## Rationale

Mechanical ventilation is an inherently dynamic process. Nonetheless, tidal volume and static tidal airway pressures (plateau, PEEP, and their difference, the driving pressure) have long served as the primary variables guiding prevention of ventilator-induced lung injury (VILI). Despite their prominence in current practice, such non-dynamic pressures cannot act alone to inflict damage; a pressure must be paired with a volume change, thereby expending energy. More specifically, any instigator of damage couples pressure applied directly to the lung, i.e., transpulmonary pressure (stress), to the associated change of lung volume (strain). Because damage depends not only on the frequency of such pairings but also on the rate of tidal stress/strain development across the epithelium and within individual extracellular fibrils that oppose lung expansion, rapid flows accentuate VILI hazard [1, 2].

Until recently, breathing frequency and duration of exposure have been underemphasized as contributors to VILI. As experimental data now indicate [3], however, more attention should be directed toward ventilating *power*, i.e., the product of energy delivered per tidal cycle and ventilating frequency, adjusted for the capacity of the lung to tolerate it. Non-mechanical factors, such as high inspired oxygen fraction, raised body temperature, and increased trans-alveolar gradients of pulmonary vascular pressure, increase vulnerability to VILI [4].

**Fig. 1:**
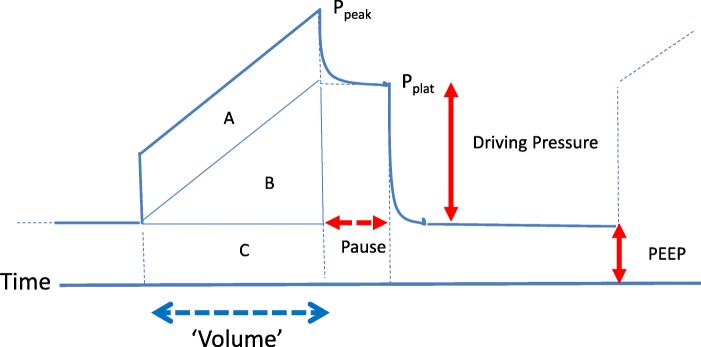
Airway pressure profile during inflation with constant flow. Under these conditions, time is an accurate surrogate for inspired volume. P_peak_ and P_plat_ are the peak dynamic and static (“plateau”) pressures. Total PEEP is comprised of the set PEEP value and auto-PEEP. Areas A, B, and C correspond to the flow resistive, tidal elastic, and PEEP-related tidal energy components, respectively. The sum of these three areas defines the total inflation energy of the tidal cycle: FR + DP/2 + PEEP_tot_. Note that while PEEP-related elastic energy (area C) elevates the inflation stress and strain platform for driving pressure, its temporarily stored elastic energy dissipates completely across the exhalation valve and circuitry during deflation

The total inflation energy of each tidal cycle is expended in overcoming flow resistive and elastic forces, both static and dynamic [5] (Fig. 1). Total energy input per minute, usually defined as “power,” is the product of minute ventilation (V_E_) and the sum of these same three tidal pressure components (flow resistive (FR = flow × resistance), DP, and PEEP). Primary emphasis is correctly placed on inflation, but expiration should not be ignored. Allowing sudden tidal deflations may accentuate VILI, perhaps due primarily to abrupt discharge of parenchymal energy [6].

Although enticing, the power hypothesis needs further refinement in order to guide VILI avoidance in clinical practice. In the “baby lung” of ARDS, for example, ventilating energy and power concentrate within a “container” with less capacity and fewer air channels to accept the gas charge [7]. This spatial concentration amplifies both the magnitude and velocity of stretching forces of the tidal breath. Moreover, the mechanically heterogeneous environment magnifies stresses at the junctions of closed and open units [8, 9]. At the bedside, relative capacity for ventilation, which reflects severity, can be roughly approximated by the ratio of measured to predicted respiratory compliance (C_obs_/C_pred_) [5].

It seems unlikely that all combinations of frequency, tidal volume, and pressure (flow resistive pressure, driving pressure, and PEEP) that sum to the same power value are equally dangerous to a given lung. Hypothetically and experimentally, a tidal strain threshold for damage initiation, however indistinct, must first be crossed [10]. That threshold level—parenchymal susceptibility to VILI—is high for intact healthy lungs and relates inversely to severity of lung disease [10]. Of the three tidal pressure constituents, the driving pressure components of energy and power relate most directly to VILI [5, 11]. Rising PEEP, though not contributing to VILI in direct proportion to its major contribution suggested by the power equation, elevates the strains associated with driving and plateau pressures closer to that injury threshold, which is lower for some lung regions than for others and decreases as VILI progresses in a positive feedback cycle. Independently of whether mode is flow or pressure regulated, both the breathing frequency and the duration of repeated exposures contribute to VILI, provided that the injuring strain threshold has been exceeded.

## Priorities in controlling damaging power

The strategy adopted during the earliest phase of support is crucial to curtailing cumulative exposure to high-stress cycles and improving eventual outcome. My first priority is to reduce vulnerability to VILI and the body’s needs for ventilation, oxygenation, and blood flow. For example, when spontaneous breathing is vigorous, deep sedation and/or paralytics minimize *transpulmonary* pressures, ventilatory demand, and the work of breathing. Estimating lung capacity serves to assess both disease severity and heterogeneity. Severely affected patients, judged either by criteria of refractory or worsening hypoxemia, or very low compliance, are proned [12]. Prone positioning usually recruits airspaces and helps even the distributions of transpulmonary pressures and tissue stresses [13]. Simultaneously, improved O_2_ exchanging efficiency typically allows reduction of FIO_2_. Fever and agitation are also suppressed to lessen ventilation demand. Successfully addressing the underlying cause of respiratory distress shortens the duration of ventilator support.

Once demands and co-contributors to vulnerability are minimized, ventilator settings assume top priority. In descending order of importance, those settings are as follows: (1) DP and PEEP, (2) V_E_, and (3) inspiration to expiration (I:E) ratio and flow profile. Whether during noninvasive or invasive ventilation, I attend first to both *transpulmonary* plateau pressure and *transpulmonary* driving pressure, and then to ventilating frequency and V_E_. I monitor the latter as a key component of power and helpful indicator of ventilating efficiency. Allowing hypercapnea (to PaCO_2_ ≤ ≈60 mmHg) promotes reductions of the frequency, V_E_, and ventilating power. I do not target a “fully open lung” but use stepwise recruiting maneuvers and “decremental” approach to find the least PEEP that achieves an effective balance between recruitment and overdistention. Assuming passive inflation and a normal chest wall, I regulate tidal volume so as to keep DP ≤ 15 cmH2O *and* plateau pressure ≤ 27 cmH_2_O. With tidal pressures and frequency set, I next modulate inspiratory flow, keeping the I:E ratio adjusted between 1:1.5 and 1:1. When the patient is passive, a controlled constant flow profile is preferred to the decelerating flow profile of pressure control [11, 14]. Because excessive power may be central to VILI risk, I assess and try to downregulate each of power’s determinants, as indicated. I do *not* set a specific upper limit for ventilating power itself, however, nor feel confident about using power criteria alone for initiating extracorporeal CO_2_ removal, as injury thresholds vary and numerical guidance from high-quality clinical studies is not currently available. (A deteriorating clinical trajectory is my primary criterion.) Finally, to minimize cumulative power exposure and VILI risk, I frequently re-evaluate my assumptions regarding needs and levels of ongoing respiratory supports.

## Data Availability

Not applicable

## Abbreviations

VILI: Ventilator-induced lung injury
PEEP: Positive end-expiratory pressure
PEEPtot: Applied PEEP + auto-PEEP
DP: Driving pressure = plateau pressure minus total PEEP
F: Mean inspiratory flow rate
R: Airway plus tissue resistance to airflow
I:E: Inspiratory to expiratory
V_E_: Minute ventilation
C_obs_: Observed respiratory compliance
C_pred_: Predicted respiratory compliance
